# Outcome of ALL With ALL-BFM-95 Protocol in Nepal

**DOI:** 10.1200/GO.22.00408

**Published:** 2023-07-10

**Authors:** Bishesh Sharma Poudyal, Bishal Paudel, Sampurna Tuladhar, Samir Neupane, Kushal Bhattarai, Utsav Joshi

**Affiliations:** ^1^ Clinical Hematology and Bone Marrow Transplant Unit, Civil Service Hospital, Kathmandu, Nepal; ^2^ Department of Pathology, Civil Service Hospital, Kathmandu, Nepal; ^3^ Department of Biochemistry, Karnali Academy of Health Sciences, Jumla, Nepal; ^4^ Department of Internal Medicine, Rochester General Hospital, New York, NY

## Abstract

**PURPOSE:**

Data on survival outcomes in patients with acute lymphoblastic leukemia (ALL) originating from Nepal are limited. We aim to present the real-world data on treatment outcomes of patients with de novo ALL treated with pediatric ALL-Berlin-Frankfurt-Muenster (BFM)-95 protocol in Nepal.

**PATIENTS AND METHODS:**

We used the medical records of 103 consecutive patients with ALL treated in our center from 2013 to 2016 to evaluate the overall survival (OS) and relapse-free survival (RFS) and analyzed the effects of clinicopathologic factors on survival outcomes in patients with ALL.

**RESULTS:**

The 3-year OS and RFS in the entire cohort was 89.4% (95% CI, 82.1 to 96.7) and 87.3% (95% CI, 79.8 to 94.7), with a mean OS and RFS of 79.4 months (95% CI, 74.2 to 84.5) and 76.6 months (95% CI, 70.8 to 82.4), respectively. Patients with prednisone good response (PGR) showed better mean OS and RFS, whereas complete marrow response on day 33 was associated with better mean OS alone. Patients with Philadelphia (Ph)-positive ALL showed worse mean RFS compared to those with Ph-negative status. On multivariate analysis, PGR (hazard ratio [HR], 0.11; 95% CI, 0.03 to 0.49; *P* = .004) and sagittal vein thrombosis (SVT; HR, 5.95; 95% CI, 1.30 to 27.18; *P* = .02) were the only independent predictors of OS and RFS, respectively. Adverse events on BFM-95 protocol included SVT (4.9%), peripheral neuropathy (7.8%), myopathy (20.4%), hyperglycemia (24.3%), intestinal obstruction (7.8%), avascular necrosis of femur (6.8%), and mucositis (46%).

**CONCLUSION:**

BFM-95 protocol appears to be a safe and effective strategy in adolescent and young adults and adult Nepalese population with ALL with a low toxicity profile.

## Teaser

The pediatric BFM protocol without laboratory monitoring of MTX is an effective and safe regimen for AYA and adult patients with ALL in Nepal.

## INTRODUCTION

Acute lymphoblastic leukemia (ALL) has shown a steady improvement in the survival outcomes over the past few decades. Although disparity exists in terms of overall survival (OS) between children, adolescents and young adults (AYAs), and older population, the 5-year event-free survival (EFS) has substantially advanced to almost 90% in the high-income countries (HICs) for childhood ALL.^1^ This is in contrast to the low- and middle-income countries (LMICs), where the OS ranges between 65% and 70% in childhood ALL, mostly because of poor socioeconomic status and limited oncological resources.^2,3^ Data published from Nepal on 300 patients of childhood ALL between 1998 and 2012 showed a 5-year EFS of 28% only.^4^ Data on survival outcome are even more limited in the adult population, and there is no evidence originating from LMICs to concur with the survival data from the developed countries. The health care system in Nepal only has a handful of medical centers in large cities which provide oncology services to the entire population. Lack of trained health workers, interrupted supply of chemotherapy and laboratory reagents, reluctance of government authorities for approval of life saving chemotherapies, financial toxicity, and scarcity of blood products have remained hindrances to advance hemato-oncology services in Nepal. Amid these adversities, the clinical hematology unit of Civil Service Hospital has been providing leukemia treatment since its establishment in 2011.

**CONTEXT**

**Key Objective**
Is it safe and effective to treat patients with acute lymphoblastic leukemia (ALL) with pediatric ALL-Berlin-Frankfurt-Muenster (BFM)-95 protocol?
**Knowledge Generated**
The 3-year overall survival and relapse-free survival in our cohort was 89.4% and 87.3%, respectively, which is in par with high-income countries. This study demonstrated a favorable toxicity profile of ALL-BFM-95 protocol, especially with methotrexate administration without rigorous monitoring of drug levels.
**Relevance**
ALL-BFM-95 protocol could be safely administered in adolescent and adult population in low- and middle-income countries with limited resources and lack of access to methotrexate level monitoring.

## PATIENTS AND METHODS

In this retrospective study, we present the real-world data on the treatment outcomes of patients with de novo ALL who were treated with pediatric Berlin-Frankfurt-Muenster (BFM)-95 intermediate risk protocol.^5^ We analyzed the medical records of 103 consecutive patients with ALL who were diagnosed and initiated on treatment in our unit between September 2013 and March 2016. The follow-up duration from the diagnosis of last patient was 3 years. We defined patients younger than 14 years as the pediatric population, 15-39 years as AYAs, and 40 years and older as adults. The following data were extracted from the medical records: age, sex, WBC count, peripheral and bone marrow blast percentage, bone marrow cytogenetics, Philadelphia (Ph) chromosome status, mixed lineage leukemia gene arrangement, prednisone response on day 8, bone marrow response on day 33, and adverse events after starting intensive chemotherapy. This study was approved by the ethical clearance committee of the Civil service hospital of Nepal.

Pediatric patients were stratified according to the ALL-BFM-95 risk stratification (Appendix 1). All pediatric patients who fulfilled the criteria for intermediate risk and all adolescent and adult patients were treated with ALL-BFM-95 intermediate risk protocol. Imatinib (400 mg dose once daily) was added from day 8 of induction chemotherapy for Ph-positive ALL. After 8 days of prednisone and one dose of intrathecal methotrexate, response to prednisone was assessed by measuring the absolute blast count in the peripheral blood. CNS disease was defined as the presence of lymphoblast in the centrifuged sample of CSF, and classification was done as per BFM-95 study.^5^ Testicular involvement was defined as a painless unilateral swelling of testis and confirmed with ultrasonography to rule out other causes. Prednisone good response (PGR) was defined as a blast count of <1 × 10^9^/L. Bone marrow morphology was evaluated for treatment response on day 33. Complete remission (CR) was defined as the presence of <5% blasts in the bone marrow with no extra medullary disease and recovery of the blood cell counts. Bone marrow relapse or refractory disease was defined as the reappearance of >5% lymphoblasts in the marrow. CNS relapse was defined as the presence of blast cells on CSF cytospinned slides while on treatment or involvement of cranial nerves or magnetic resonance imaging findings and histopathological evidence of parenchymal involvement after achieving CR.^5^ Induction death included any event of death during induction chemotherapy before the recovery of blood count. Combined relapse comprised recurrence of disease in both bone marrow and extra medullary site(s). Relapse-free survival (RFS) and OS were calculated from the date of diagnosis until patient shows evidence of disease relapse or death, respectively.

All patients received high-dose methotrexate without dose modification (3 g/m^2^ for B-cell ALL and 5 mg/m^2^ for T-cell ALL once every 15 days for 2 months), and all received complete doses of methotrexate according to the ALL-BFM-95 protocol. Serum methotrexate level could not be monitored as per protocol because of unavailability of the test in our center. The treatment team would ensure that the patient had a good urine output up to at least 72 hours from the initiation of methotrexate infusion by giving adequate intravenous hydration. A target urinary pH > 7 was maintained at least from the 4-hour mark since methotrexate infusion up to at least 72 hours by infusing intravenous sodium bicarbonate. A strict intake and output charting was done for accurate assessment of fluid balance and volume status. If patients had a net positive fluid balance of >400 mL/m^2^/12 hours, intravenous furosemide was administered at a dose of 0.5 mg/kg as needed (maximum dose: 20 mg [prn]). Intravenous leucovorin rescue was started at a dose of 15 mg/m^2^ at 42, 48, 54, 60, and 66 hours of initiation of metrotrexate infusion. Patients were carefully monitored for any signs of toxicity, especially evaluating for oliguria/anuria, hypertension, edema, weight gain, emesis, confusion, blurred vision, or significant elevation of serum creatinine which could all be the manifestations of slow elimination of methotrexate. If these clinical findings were identified, forced diuresis, urinary alkalinization, and two more doses of leucovorin were repeated, and a stricter monitoring of volume status, vital signs, and serum chemistry was done.

OS and RFS were assessed using Kaplan-Meier method and compared between groups using the log-rank test. Using backward step selection, multivariate Cox proportional hazard regression analysis incorporating factors with *P* < .1 in univariate analysis was used to evaluate independent factors associated with OS and RFS. Logistic regression analysis was separately performed to evaluate factors associated with PGR and bone marrow response on day 33. All analyses were performed using SPSS version 22 (IBM SPSS Statistics for Windows, Armonk, NY).

### Ethics Statement

We have obtained institutional review board approval and have obtained informed consent from each participant or each participant's guardian.

## RESULTS

### Baseline Characteristics of the Study Population

Of the 103 patients included in the study, 61% were males, and the median age was 22 years (range, 9-77 years). Nine patients (8.7%) were younger than 14 years, and only three patients were 60 years and older. The median WBC count was 18.5 × 10^9^/L (range, 0.2-700 × 10^9^/L), and the median blast percent was 66% (range, 32-98). Only 10.7% of the patients were Ph-positive, 71.8% showed PGR on day 8, and 70.9% showed complete marrow response on day 33 (Table 1). Only two patients had testicular involvement, and one patient showed CNS involvement at the time of diagnosis. Four patients were lost to follow-up until 2019.

**TABLE 1:**
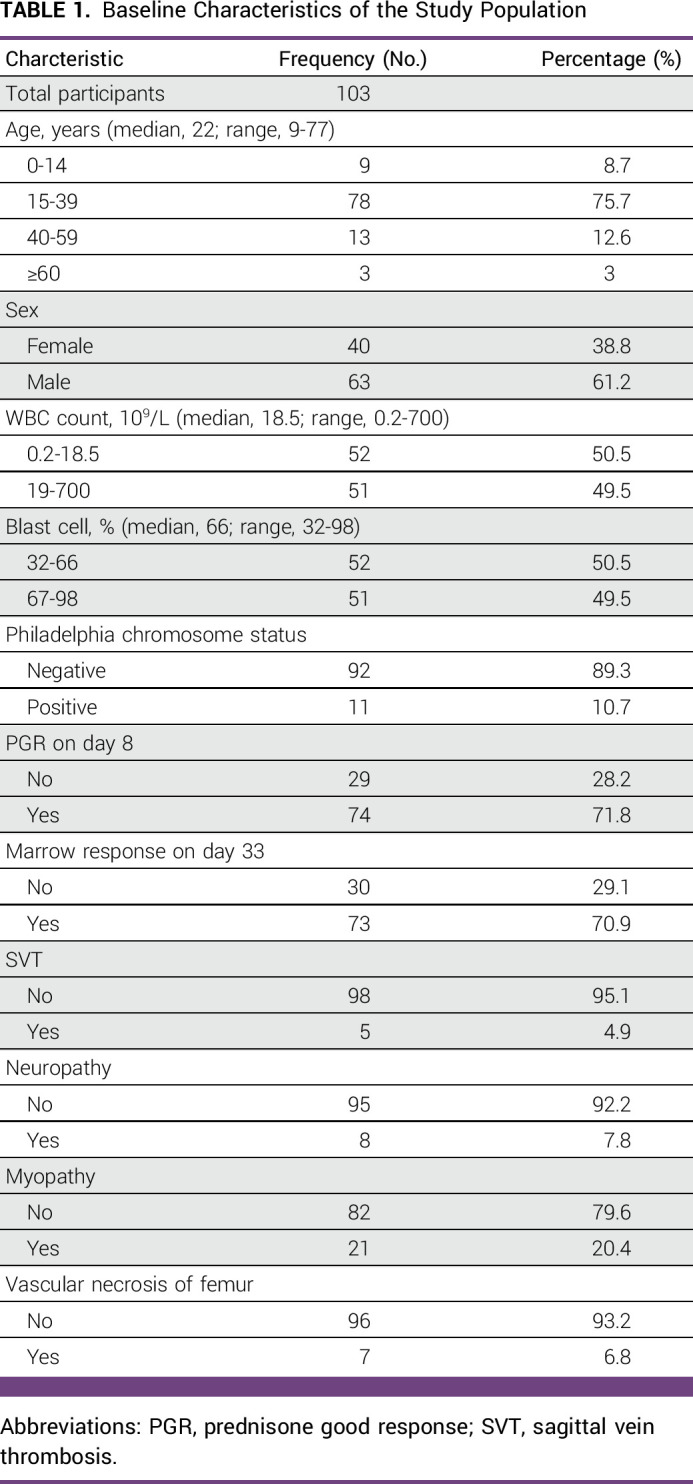
Baseline Characteristics of the Study Population

### Adverse Events

Adverse events in our study population included sagittal vein thrombosis (SVT) in five patients (4.9%), peripheral neuropathy in eight patients (7.8%), myopathy in 21 patients (20.4%), steroid-induced hyperglycemia in 25 patients (24.3%), vincristine-induced subacute intestinal obstruction in eight patients (7.8%), avascular necrosis of femur in seven patients (6.8%), and acute pancreatitis in one patient (0.9%). Grade 3-4 mucositis was present only in 19% of our patients.

### Survival Outcome in Overall Population

**FIG 1:**
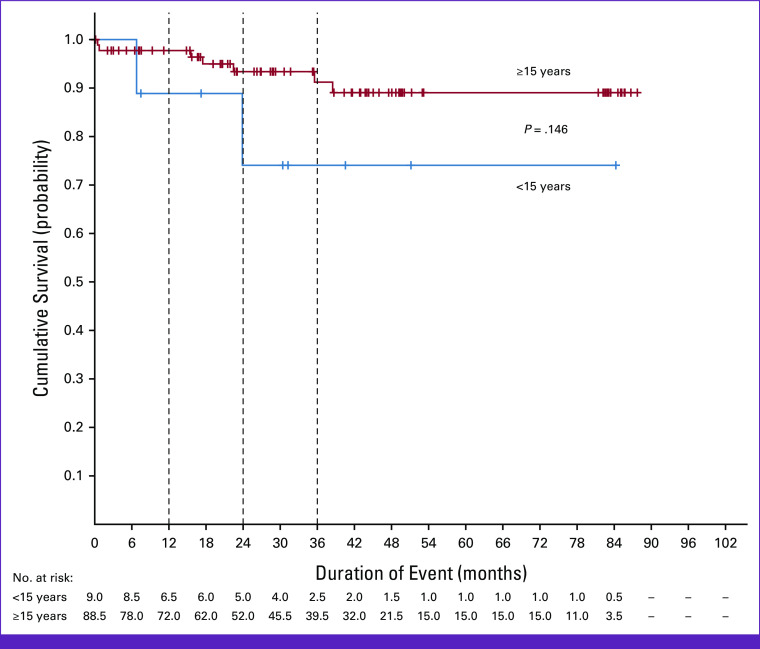
Overall survival on the basis of age group.

**FIG 2:**
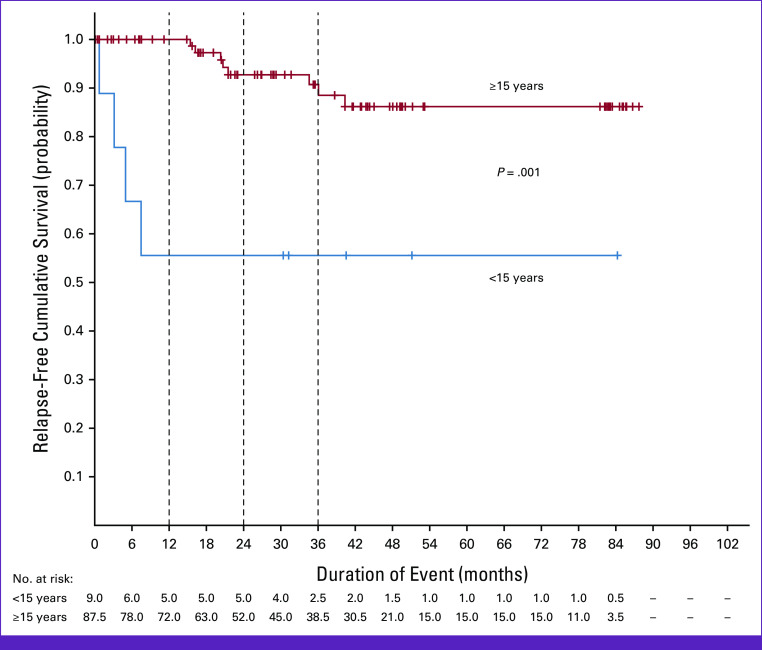
Relapse-free survival on the basis of age group.

The 3-year cumulative survival and RFS in the entire cohort was 89.4% (95% CI, 82.1 to 96.7) and 87.3% (95% CI, 79.8 to 94.7), respectively. In patients older than 14 years, the 3-year cumulative survival and RFS was 91.2% (95% CI, 84.3 to 98.1) and 90.7% (95% CI, 83.4 to 97.9), respectively (Figs 1 and 2). The mean OS and RFS was estimated to be 79.4 months (95% CI, 74.2 to 84.5) and 76.6 months (95% CI, 70.8 to 82.4), respectively (Appendix Table A1). Among patients 60 years and older, two tolerated full course of chemotherapy and were alive at the time of last follow-up, whereas one died after 7 months of initiation of treatment because of neutropenic sepsis.

### Prognostic Factors for OS and RFS

There was no significant difference in terms of mean OS on the basis of age (0-14 *v* ≥15; *P* = .15), sex (*P* = .68), WBC count (≤18.5 *v* >19; *P* = .34), blast percent (≤66 *v* ≥67; *P* = .07), and Ph chromosome status (*P* = .85). Patients with PGR showed better mean OS compared with those with poor prednisone response (84.1; 95% CI, 80.0 to 88.1 *v* 56.7; 95% CI, 39.4 to 74.0; *P* < .001). Complete marrow response on day 33 was associated with better mean OS compared with those who did not achieve CR (81.9; 95% CI, 77.1 to 86.8 *v* 68.1; 95% CI, 54.8 to 81.4; *P* = .04). In terms of mean RFS, there was no significant difference on the basis of sex (*P* = .20), WBC count (≤18.5 *v* >19; *P* = .09), blast percent (≤66 *v* ≥67; *P* = .68), and marrow response on day 33 (*P* = .51). Ph-positive patients showed worse mean RFS compared with Ph-negative patients (37.0; 95% CI, 25.8 to 48.2 *v* 79.3; 95% CI, 73.9 to 84.8; *P* = .004). Parallel to mean OS, PGR was also associated with better mean RFS (79.6; 95% CI, 74.0 to 85.3 *v* 59.3; 95% CI, 42.0 to 76.5; *P* = .01). The presence of SVT correlated to worse mean RFS (34.2; 95% CI, 21.6 to 46.7 *v* 78.9; 95% CI, 73.4 to 84.3; *P* = .003; Appendix Table A2).

### Multivariate Analysis for OS and RFS

PGR and marrow response on day 33 were included in the multivariate analysis for OS, and age, Ph chromosome status, PGR, and SVT were included in the analysis for RFS. PGR was found to be an independent predictor of OS (hazard ratio [HR], 0.11; 95% CI, 0.03 to 0.49; *P* = .004), whereas SVT was the only independent predictor of RFS (HR, 5.95; 95% CI, 1.30 to 27.18; *P* = .02). Although PGR and Ph-negative status were associated with better RFS, this did not reach statistical significance (Tables 2 and 3).

**TABLE 2:**
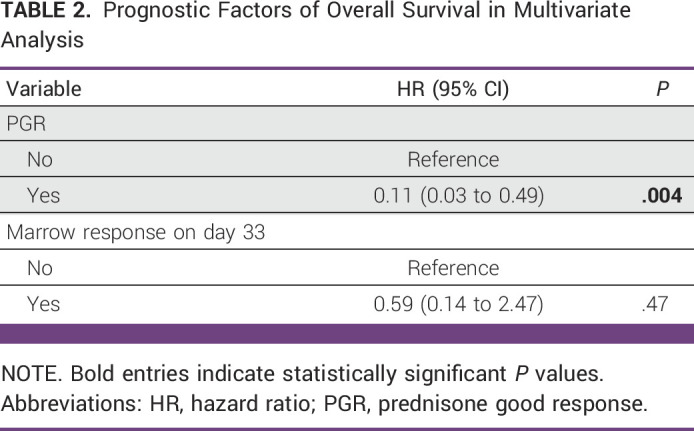
Prognostic Factors of Overall Survival in Multivariate Analysis

**TABLE 3:**
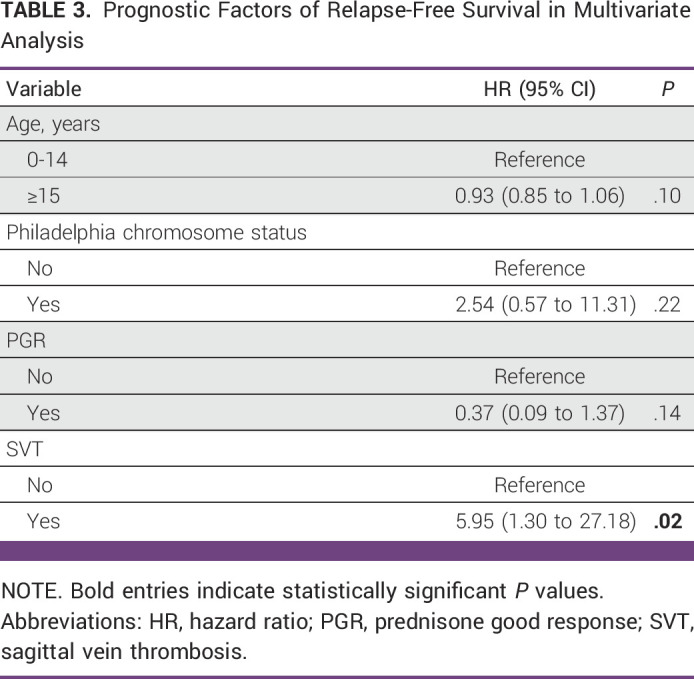
Prognostic Factors of Relapse-Free Survival in Multivariate Analysis

## DISCUSSION

ALL-BFM-95, a widely used protocol for pediatric ALL, has also revolutionized the treatment of ALL in adolescents and adults. Compared with the traditional adult ALL protocol, cure rate has almost doubled with BFM-95 protocol in AYA, although results have been less encouraging because of heightened toxicity in adults older than 55 years.^6-8^ These results inspired us to use the ALL-BFM-95 protocol in our population of adolescents and adults. The 3-year OS of 89.4% and mean OS of 6.6 years are comparable with the data published from HIC.^9^ Reasons for higher survival rate in our cohort could be greater experience of treating patients with acute leukemia (>120 patients with acute leukemia every year) resulting in a low induction mortality rate, greater proportion of young patients, smaller number of Ph-positive patients, wider availability of antimicrobials and blood products, and better supportive care. Although our study showed relatively poor survival outcomes in the pediatric cohort, the small sample (8.7%) makes it difficult to draw any definite conclusion and compare the result with AYAs which comprises the majority of patients in this cohort (75.7%) based on our referral pattern.

The difference in the RFS between Ph-positive and Ph-negative ALL despite the use of imatinib is alarming, and we presume this is secondary to higher number of CNS relapse in Ph-positive patients. However, this difference did not translate to statistical significance in multivariate analysis, likely because of the small size of Ph-positive cohort and low power. Dasatinib is known to have greater CNS penetration than imatinib and thereby, shows better RFS than imatinib in Ph-positive ALL.^10^ On the basis of our findings of high incidence of CNS relapse in Ph-positive ALL, it seems we should consider replacing imatinib with dasatinib in our patients with Ph-positive ALL.

PGR and complete marrow response on day 33 has been shown to be a favorable prognostic factor for survival and also an indicator of treatment response.^11,12^ Our study also showed a stark difference in the 3-year OS (95.7% *v* 62.5%) and RFS (91.9% *v* 65.5%) among patients with good versus poor prednisone response, although this was only significant for OS in multivariate analysis. The finding of SVT as a poor predictor of RFS was interesting and likely correlates with the delay in treatment administration after the diagnosis. Per protocol, patients diagnosed with cerebral venous thrombosis were carefully monitored in the intensive care unit, and their treatment postponed until clinical improvement. In addition, the poor RFS in this study is consistent with the GRAALL experience which showed lower 3-year OS and disease-free survival in patients with cerebral venous thrombosis, albeit this was nonsignificant.^13^

In the past, hematologists in LMICs were reluctant to use the pediatric-inspired protocol such as ALL-BFM-95 in the adult population because of the fear of increased toxicities. In our study, common toxicities in terms of hyperglycemia, myositis, pancreatitis, avascular necrosis, peripheral neuropathy, sepsis, fungal pneumonia, and venous thrombosis were quite comparable with the published literature.^14^ Grade 3-4 mucositis of 19% in our population without testing serum methotrexate level supports the notion that adequate hydration, careful monitoring of urine alkalization/diuresis, and clinical judgment is as important as laboratory investigation.

We believe that the lack of availability of data on minimal residual disease (MRD) is one of the major drawbacks of our study. However, physicians in LMICs need to be very careful while copy-pasting the treatment guidelines that could lead to unprecedented increase in the cost of treatment. MRD monitoring has been incorporated in most of the treatment protocols, but patients may still relapse after obtaining MRD negativity. Financial toxicities related to treatment of acute leukemia are reported astonishingly high in Nepal. Therefore, more studies are needed to incorporate MRD monitoring in low-income settings because of the need for frequent monitoring and expenses related to it.

Given that our study only had three patients 60 years and older and majority were AYAs, we have to consider the possibility of healthy patient bias contributing to this high OS and RFS. We were also unable to include data on ABL1 kinase mutation in patients with relapsed Ph-positive ALL because of technical and financial constraints. With the outbreak of COVID-19, follow-up of this cohort was compromised, and data beyond 2019 have not been included in this study because of the risk of selection bias. Although a referral center for acute leukemia, ours is not a primary referral institution for pediatric population, and the small pediatric cohort likely resulted in a fallaciously poor outcome. Despite including each consecutive patient diagnosed and treated at our center, there remains a possibility of sampling bias with a retrospective study like ours. Patient medical record documentation is rigorous in our center with a stringent protocol in place to document chemotherapy administered, dosage and timing, daily laboratory results, and adverse events while on chemotherapy, minimizing the proportion of missed events. Nevertheless, in a LMIC such as Nepal, conventional techniques and good clinical judgment still play a paramount importance to reduce the cost of treatment rather than relying on expensive investigation.

In conclusion, the result of our study shows the ALL-BFM-95 protocol to be safe and effective in AYAs and adult Nepalese population with ALL with a low toxicity profile. Future studies should consider replacing imatinib with dasatinib in newly diagnosed Ph-positive ALL to mitigate CNS relapse rate and also explore the possibility to conduct clinical trials in the field.

## Figures and Tables

**TABLE A1:**
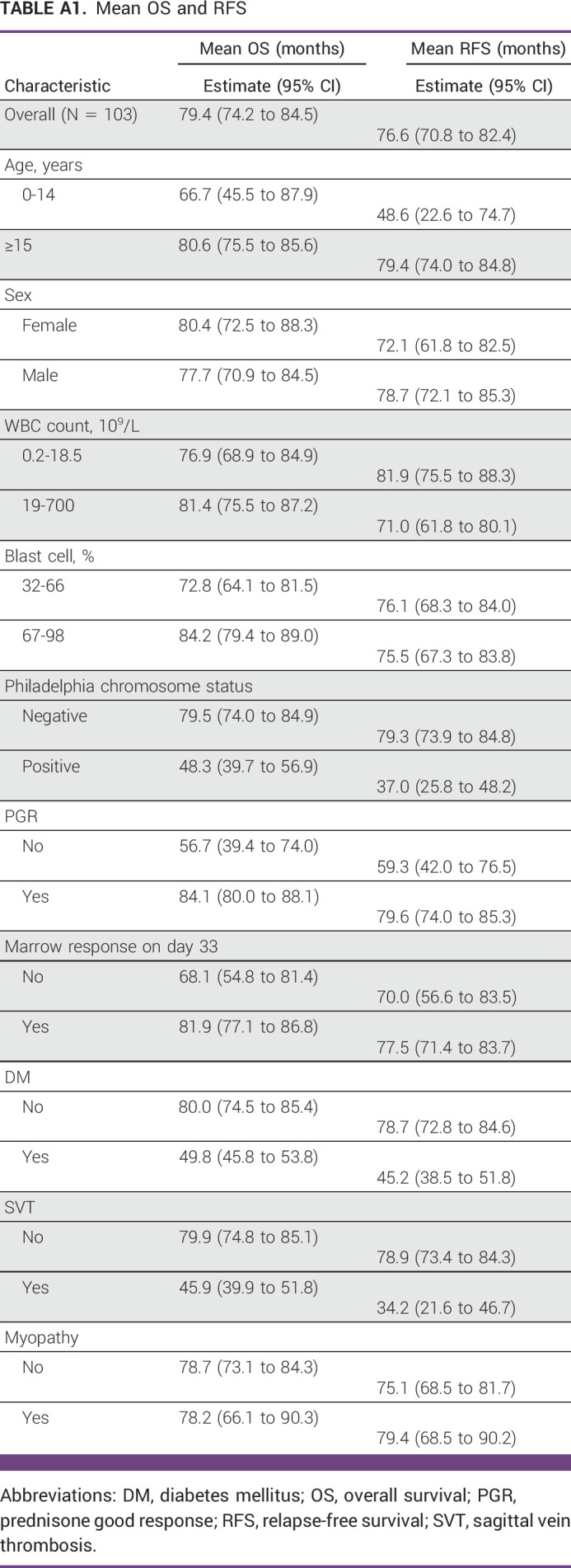
Mean OS and RFS

**TABLE A2:**
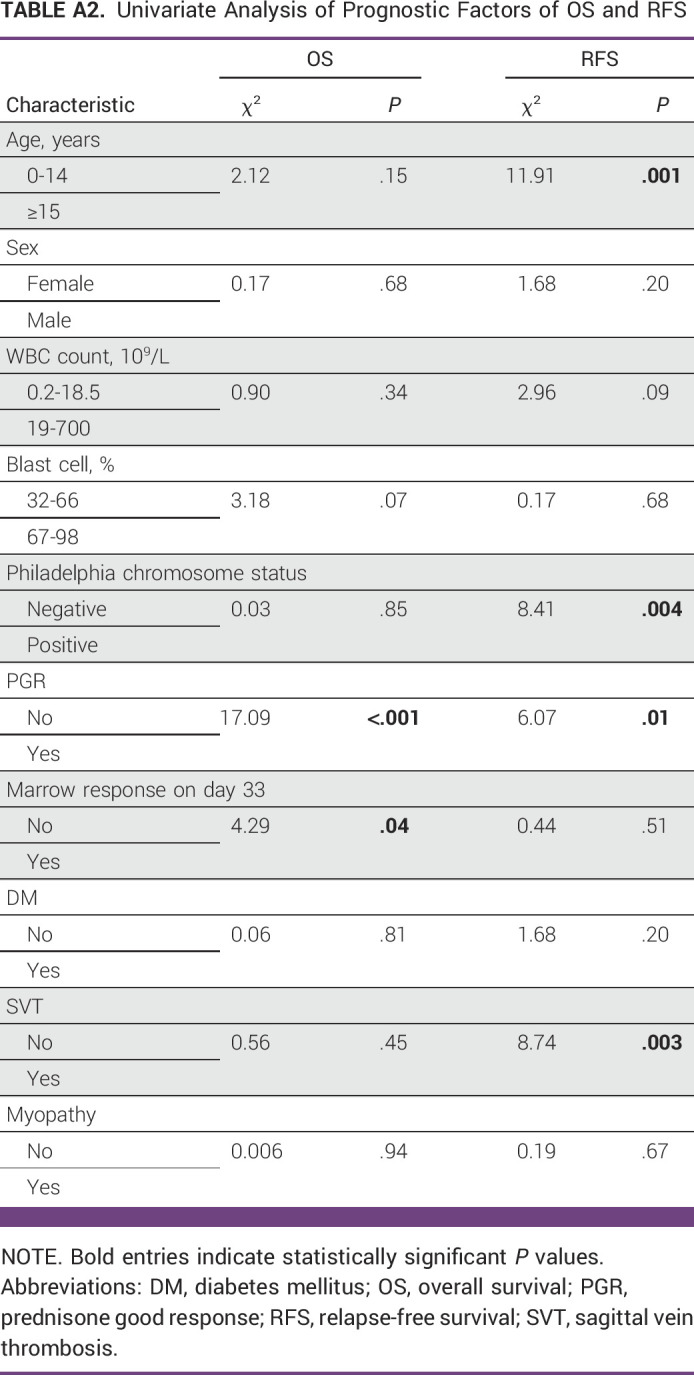
Univariate Analysis of Prognostic Factors of OS and RFS

## APPENDIX 1. Supplementary Method

The risk stratification criteria as per ALL-BFM-95 protocol was based on age, WBC at diagnosis, immunophenotype, prednisone response, response to induction treatment, and unfavorable translocations t(9;22) and t(4;11).

1. High risk—prednisone poor response and/or no complete remission on day 33 and/or evidence of t(9;22) (or breakpoint cluster region (BCR)/Abelson (ABL)) and/or evidence of t(4;11) (or mixed lineage leukemia/AF4).
2. Medium/intermediate risk—no high risk criteria and initial WBC 20 × 10^9^/L or more and/or age at diagnosis <1 or 6 years or older, t(1:19), Pro B-cell acute lymphoblastic leukemia (B-ALL) without 11q23 abnormality, CNS disease or testicular disease at diagnosis, and/or T-cell acute lymphoblastic leukemia (T-ALL).
3. Standard risk—no high risk or intermediate risk criteria and initial WBC <20 × 10^9^/L and age at diagnosis between 1 and 6 years, steroid good response, post induction marrow in remission, and no T-ALL.
